# Comparison of the Nanostructure and Mechanical Performance of Highly Exfoliated Epoxy-Clay Nanocomposites Prepared by Three Different Protocols

**DOI:** 10.3390/ma7064196

**Published:** 2014-05-30

**Authors:** Fatemeh Shiravand, John M. Hutchinson, Yolanda Calventus, Francesc Ferrando

**Affiliations:** 1 Centre for NanoEngineering, Departament de Màquines i Motors Tèrmics, Escola Tècnica Superior d’Enginyeries Industrial i Aeronàutica (ETSEIAT), Universitat Politècnica de Catalunya, Terrassa 08222, Barcelona, Spain; E-Mails: fatemeh.shiravand@mmt.upc.edu (F.S.); calventus@mmt.upc.edu (Y.C.); 2 Department of Mechanical Engineering, Universitat Rovira i Virgili, C/Països Catalans 26, Tarragona 43007, Spain; E-Mail: f.ferrando@urv.cat

**Keywords:** nanocomposite, exfoliation, pre-conditioning, mechanical properties, homopolymerisation, epoxy, layered silicate, differential scanning calorimetry, nanostructure, montmorillonite

## Abstract

Three different protocols for the preparation of polymer layered silicate nanocomposites based upon a tri-functional epoxy resin, triglycidyl *para*-amino phenol (TGAP), have been compared in respect of the cure kinetics, the nanostructure and their mechanical properties. The three preparation procedures involve 2 wt% and 5 wt% of organically modified montmorillonite (MMT), and are: isothermal cure at selected temperatures; pre-conditioning of the resin-clay mixture before isothermal cure; incorporation of an initiator of cationic homopolymerisation, a boron tri-fluoride methyl amine complex, BF_3_·MEA, within the clay galleries. It was found that features of the cure kinetics and of the nanostructure correlate with the measured impact strength of the cured nanocomposites, which increases as the degree of exfoliation of the MMT is improved. The best protocol for toughening the TGAP/MMT nanocomposites is by the incorporation of 1 wt% BF_3_·MEA into the clay galleries of nanocomposites containing 2 wt% MMT.

## 1. Introduction

Epoxy resins constitute a well-known family of thermosetting polymers that have been used widely and over a period of many years in various industrial fields such as coatings, high-performance adhesives and other engineering applications, because of their many desirable properties. However, the cured epoxy resin exhibits low fracture toughness, poor resistance to crack initiation and growth, and low impact strength. Many studies have been made over the past decades to find ways for toughening the epoxy matrix, for example by the addition of a second phase such as rigid or elastomeric particles. More recently, nanoclay fillers, which have both a high aspect ratio (200–1000) and high modulus (170 GPa), have emerged as a new approach for the improvement of the mechanical performance, including the toughness and impact resistance, of the cured epoxy matrix [1,2,3]. Furthermore, these clays have attracted considerable attention because of their low cost and ready availability.

In addition, the possibility of achieving with nanoclays the same degree of reinforcement as obtained with microscopic fillers but with a much lower filler content is particularly attractive for applications in which the density of the composite materials is important, for example in the aerospace and automotive industries. This requires that the idealised structure of the polymer layered silicate nanocomposite be developed from that of a conventional composite or microcomposite, in which the clay acts as a conventional filler, to that of an exfoliated nanocomposite, in which the individual clay layers are distributed uniformly in the matrix in order to maximise the surface contact between clay and matrix and to inhibit the propagation of cracks. In essence, this has been the goal of much of the research in this area in recent years, and is also addressed in the present paper.

The unmodified nanoclay, such as montmorillonite (MMT), consists of stacks of silicate layers, each of the order of 1 nm thick, and is naturally hydrophilic and hence unsuitable for use as a filler in most of the commonly used epoxy resins, which are hydrophobic. Consequently, the montmorillonite clay is commonly modified organically by an ion exchange process in which the cations are replaced by, for example, quaternary ammonium ions with long alkyl chains. This not only makes the clay organophilic but also increases the spacing between the clay layers, which facilitates the intercalation of the epoxy resin in the preparation of the epoxy-clay nanocomposites. In order to accomplish the desired improvement in mechanical properties of these nanocomposites, it is necessary for the clay layers to be further separated and dispersed homogeneously throughout the polymer matrix, in a process referred to as exfoliation [4,5], when the epoxy-clay mixture is cured. However, achieving a highly exfoliated nanostructure in these epoxy layered silicate nanocomposites has proven difficult, and it is common to refer to “partial” exfoliation or to consider the nanostructure to be exfoliated when the *d*-spacing of the clay layers cannot be detected by Small Angle X-ray Scattering (SAXS), which normally implies layer separations greater than about 8 nm [6,7,8,9,10]. Very large layer separations is not the only reason for the absence of diffraction peaks, however, as has been noted by several authors [11,12,13], and consequently it is important that nanostructural characterisation by Transmission Electron Microscopy (TEM) should also be made.

For epoxy-clay nanocomposites, it has been suggested that, in order to maximise the degree of exfoliation, the intra-gallery epoxy homopolymerisation reaction which takes place between the clay layers should occur before the extra-gallery cross-linking reaction of the epoxy resin with the curing agent [14,15,16]. This hypothesis is supported by some observations made for the epoxy-clay system based upon the bi-functional epoxy diglycidyl ether of bisphenol-A (DGEBA) and organically modified montmorillonite [17]. When this system was cured with a polyoxypropylene diamine, a marked shoulder appeared on the high temperature flank of the exothermic cure reaction, indicating that a reaction, interpreted as being the intra-gallery homopolymerisation, was occurring after the extra-gallery cross-linking reaction. As a consequence, the cured nanocomposite in this case was poorly exfoliated, indicated by the existence of scattering peaks in the SAXS results. When the same epoxy-clay system was cured with an –NH_2_ terminated hyperbranched polyethyleneimine, however, this shoulder was not present, suggesting that the intra-gallery reaction was occurring simultaneously with the cross-linking reaction [18]. At the same time, the SAXS results showed no scattering peaks, suggestive of a much better degree of exfoliation.

In contrast, for layered silicate nanocomposites based on a tri-functional epoxy resin, triglycidyl *para*-amino phenol (TGAP), and with the same MMT clay, two distinct reactions were observed in the isothermal cure reaction with 4,4-diamino diphenyl sulphone (DDS) as the curing agent: the first was attributed to the epoxy homopolymerisation reaction taking place within the clay galleries, while the second was attributed to the bulk cross-linking reaction in the extra-gallery regions [19,20]. The justification for the assignments of these reactions is considered further in the present paper. On the basis of the above hypothesis, therefore, this system appears to afford a mechanism for improved exfoliation in these epoxy-clay nanocomposites, and in earlier work we investigated separately the effect of the preparation procedure on the nanostructure of these cured nanocomposites. The different preparation procedures used were: (i) pre-conditioning of the resin-clay mixture before the addition of the curing agent [19]; (ii) suitable selection of the isothermal cure temperature [20]; and (iii) incorporation of an initiator of cationic homopolymerisation within the clay galleries [21].

In the present work, for each of these different preparation procedures, for which the results have been presented separately in earlier publications [19,20,21], we make a comparison of the cure reaction kinetics, monitored by differential scanning calorimetry (DSC), and the degrees of exfoliation as identified by nanostructural characterisation techniques such as Small Angle X-ray Scattering (SAXS) and Transmission Electron Microscopy (TEM), in order to clarify the mechanisms responsible and the preparation conditions required for the exfoliation of these epoxy polymer layered silicate nanocomposites. In addition, we further include the results of new experiments which show how the enhancement in the impact strength of the cured nanocomposites can be correlated with the improved exfoliation in the nanostructure.

## 2. Experimental

### 2.1. Materials

The epoxy resin, TGAP, with trade name Araldite MY0510 (Huntsman Advanced Materials, Everburg, Belgium) and an epoxy equivalent between 95 and 106 g/eq, the curing agent, 4,4-diamino diphenyl sulphone (DDS), with trade name Aradur 976-1 (Huntsman Advanced Materials, Everburg, Belgium), the organically modified montmorillonite (MMT), with trade name Nanomer I.30E (Nanocor Inc., Hamburg, Germany) consisting of 70–75 wt% montmorillonite and 25–30 wt% octadecylamine, with a cation exchange capacity (CEC) of 92 meq/100 g, and the boron trifluoride monoethylamine complex, BF_3_·MEA, (Sigma-Aldrich, Steinheim, Germany) as the cationic initiator for the homopolymerisation reaction, were all used without further purification.

### 2.2. Moulds

Samples for the mechanical tests were cured in open moulds which had been machined from Teflon. For the dynamic mechanical analysis, the mould consisted of a cylindrical block with four cylindrical cavities, approximately 10 mm diameter and 11 mm in depth, with a slight taper to facilitate the removal of the cured samples. For the impact tests, the mould consisted of a rectangular block with nine separate rectangular cavities, approximately 25 mm × 12 mm × 2.5 mm. In each case, the block was clamped to a rigid base-plate (circular for the dynamic mechanical tests, rectangular for the impact tests) which could be removed in order to extract the cured samples. The cured samples could be removed from the moulds without the need for the previous application of any release agent. Each of these moulded samples was then machined to the required final dimensions for the different mechanical tests.

Samples for transmission electron microscopy were cast into a flat embedding mould made of silicone rubber and with overall dimensions 105 mm × 70 mm × 7 mm deep. This mould contained 24 numbered tapered troughs, 6 mm × 13 mm × 3 mm deep, designed such that the sample is easily accommodated into the ultramicrotome holder.

### 2.3. Sample Preparation

**Figure 1:**
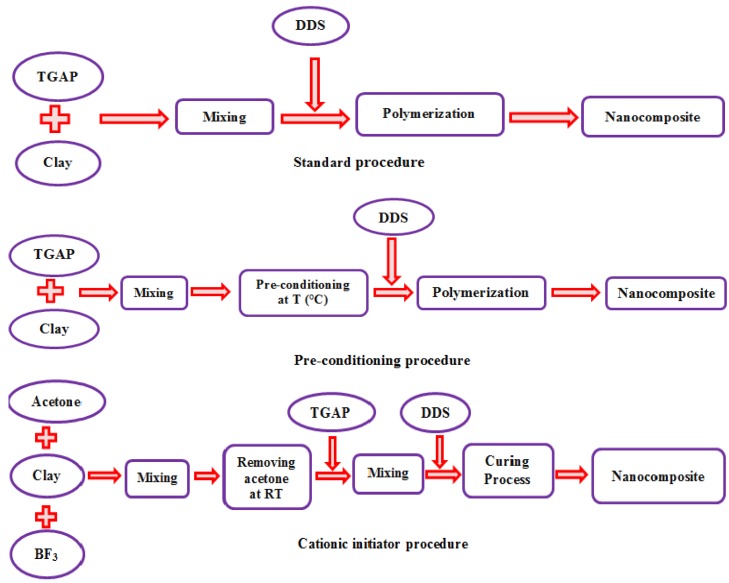
Schematic illustration of the different nanocomposite preparation procedures.

The preparation of the nanocomposites was made according to three different procedures, which are detailed as follows and are illustrated schematically in Figure 1.

#### 2.3.1. Standard Procedure

For the standard procedure, the TGAP epoxy resin and the clay were mixed together by mechanical stirring at a speed of ~200 rpm for approximately 4 h. The proportions of clay used were 2 wt% and 5 wt% MMT with respect to the TGAP weight. The curing agent, DDS, was then added to the TGAP/MMT mixture in a proportion of 52 wt% and mixed by hand for 5–7 min on a hot-plate at 80 °C. This proportion of DDS corresponds to an excess molar epoxy ratio (1:0.85), as recommended by the resin manufacturer. The mixture was finally degassed in a vacuum chamber (Heraeus RVT360, W.C.Heraeus GmbH, Hanau, Germany) for approximately 10 min at room temperature and 100 Pa pressure, and the mixture was then either poured into the appropriate mould for the subsequent machining of the mechanical test samples or used for taking small samples for scanning in the DSC. For the mechanical test samples, the standard cure schedule consisted of two isothermal steps in an air-circulating oven: first at 150 °C for 3 h or at 180 °C for 1.5 h, followed by a post-cure at 210 °C for 2 h.

#### 2.3.2. Pre-conditioning Procedure

For the pre-conditioned samples, mixtures of TGAP epoxy resin and either 2 wt% or 5 wt% MMT were mixed by mechanical stirring and were then stored at either room temperature (RT) or at a higher temperature (40 °C, 60 °C, 80 °C), using a thermostatic bath (Techne, TE-8D, Labequip, Ontario, Canada). In the present work, we show the results for samples pre-conditioned by storage at 40 °C for 78 and 67 days, for proportions of 2 wt% and 5 wt% MMT, respectively. Following this pre-conditioning treatment, the required amount of DDS was added to the TGAP/MMT mixtures, according to the epoxy equivalent (*EE*) of the TGAP, determined by titration [22], in order to give an excess epoxy ratio (mass ratio 1:0.52). The mixture was then degassed under vacuum at room temperature as above, poured into the moulds, and subjected to the same cure schedule as described above for the standard procedure.

#### 2.3.3. Cationic Initiation Procedure

For the samples in which cationic homopolymerisation is initiated in the clay galleries, the MMT and BF_3_·MEA were first mixed together, using acetone as a solvent, in various proportions such as to give 0.5 wt% and 1 wt% of BF_3_·MEA and 2 wt% and 5 wt% of MMT, both with respect to the TGAP content, in the final nanocomposite. The acetone was then removed by evaporation at room temperature over a period of about one day. The resulting mixture, ground to a fine powder, was then dispersed in the TGAP by high shear mechanical mixing (Polytron, PT1200C, Kinematica AG, Lucerne, Switzerland) at room temperature before being degassed under vacuum as above. This TGAP/MMT/BF_3_·MEA mixture was then heated on a hot-plate to 80 °C and the DDS was added to give an excess epoxy ratio (mass ratio 1:0.52). Finally, the mixture was poured into moulds as required and cured according to the following schedules, different from those used for the samples prepared by the standard and pre-conditioning procedures: (i) 110 °C for 1 h, 125 °C for 6 h; (ii) 100 °C for 2.5 h, 150 °C for 2 h; (iii) 100 °C for 2.5 h, 180 °C for 1 h. Each of these cure schedules was followed by a post-cure at 210 °C for 2 h.

Samples were prepared according to one or more of the above procedures for a variety of purposes: to follow and analyse the curing kinetics by DSC; to examine the nanostructure of the cured nanocomposites by SAXS and TEM; and to determine the dynamic mechanical properties and, in particular, the impact strength of the cured nanocomposites in order to see whether the impact strength can be correlated with the nanostructure. A summary of the various compositions that were prepared specifically for the purposes of the impact testing and based on these procedures is given in Table 1.

It should be noted that not all of these samples listed in Table 1 were studied here by DSC, SAXS and TEM; for those that were used here for these purposes, their composition and preparation conditions will be clearly stated when the results are discussed in a later section.

**Table 1:** Sample identification for different compositions and preparation conditions of TGAP/clay nanocomposites prepared for impact testing.

Composition (numbers in brackets represent wt%)	Cure profile (°C)			Sample identification
	Step 1	Step 2	Step 3	
TGAP/DDS(52)	150	–	210	no clay; reference
TGAP/MMT(2)/DDS(52)	150	–	210	2%, 150 °C
TGAP/MMT(2)/DDS(52)	180	–	210	2%, 180 °C
TGAP/MMT(2) + BF_3_·MEA(0.5)/DDS(52)	110	125	210	2% + 0.5% BF_3_, 125 °C
TGAP/MMT(2) + BF_3_·MEA(0.5)/DDS(52)	100	150	210	2% + 0.5% BF_3_, 150 °C
TGAP/MMT(2) + BF_3_·MEA(0.5)/DDS(52)	100	180	210	2% + 0.5% BF_3_, 180 °C
TGAP/MMT(2) + BF_3_·MEA(1)/DDS(52)	110	125	210	2% + 1% BF_3_, 125 °C
TGAP/MMT(2)/DDS(52), pre-conditioned 40 °C, 78 days	150	–	210	2%, pre 40 °C
TGAP/MMT(5)/DDS(52)	150	–	210	5%, 150 °C
TGAP/MMT(5)/DDS(52)	180	–	210	5%, 180 °C
TGAP/MMT(5) + BF_3_·MEA(0.5)/DDS(52)	110	125	210	5% + 0.5% BF_3_, 125 °C
TGAP/MMT(5) + BF_3_·MEA(1)/DDS(52)	110	125	210	5% + 1% BF_3_, 125 °C
TGAP/MMT(5)/DDS(52) pre-conditioned 40 °C, 67 days	150	–	210	5%, pre 40 °C

### 2.4. Differential Scanning Calorimetry (DSC)

The calorimetric curing experiments were carried out using a conventional DSC (DSC821e, Mettler-Toledo, Schwerzenbach, Switzerland) equipped with a sample robot and Haake EK90/MT intracooler (Thermo Electron Corporation GmbH, Karlsruhe, Germany). All experiments were performed with a flow of dry nitrogen gas at 50 mL/min. The data evaluation was performed with the STAR^e^ software, and the instrument was calibrated for both heat flow and temperature using indium. Sample masses were typically in the range 8–10 mg, weighed on a microbalance and encapsulated in sealed standard aluminium pans with a lid. After mixing, samples were immediately inserted into the DSC furnace, which was previously heated to the appropriate isothermal cure temperature, whereupon the curing experiment was immediately started. The isothermal cure experiments were made at the various temperatures indicated in Table 1 above.

For the calorimetric determination of the glass transition temperature, a stochastic temperature modulated DSC method, TOPEM (Mettler Toledo DSC823e, Mettler Toledo AG Analytical, Schwerzenbach, Switzerland), was used [23,24]. The TOPEM instrument was equipped with an intracooler (Julabo FT400, Julabo GmbH, Seebach, Germany) and was calibrated for both heat flow and temperature using indium. Scans were made over a temperature range from 100 to 285 °C at 2 K/min, with temperature pulses of amplitude ±0.5 °C and a switching time range of 15–30 s, and with a flow of dry nitrogen gas at 50 mL/min. Sample masses for this technique were typically in the range of 10–15 mg, encapsulated in sealed standard aluminium pans with a lid. The glass transition temperatures were determined from the sigmoidal change in the so-called quasi-static specific heat capacity, *c*_p0_.

### 2.5. Nanostructural Characterisation

Small angle X-ray scattering (SAXS) and transmission electron microscopy (TEM) were both used to characterise the nanostructure of the nanocomposites. X-ray diagrams were obtained using a Bruker D8 Advanced diffractometer (Bruker Corporation, Billerica, MA, USA), measurements being taken in a range of 2θ = 1°–8° with copper Kα radiation (λ = 0.1542 nm). The power settings were 50 kV and 40 mA. For all the samples, the scans were made with steps in 2θ of 0.02° and with a time of 10 s for each step. The intercalation of the epoxy resin into the clay galleries was determined for the resin-clay mixtures in the form of viscous liquids; the sample was spread in a thin layer on the sample holder, and the viscosity is such that no significant settling of the clay layers will take place during the scan. For the cured nanocomposites, the scattering diagrams were obtained for samples in the form of powder after ball-milling (Retsch model MM 400, Retsch GmbH, Haan, Germany) the bulk samples using 20 mm diameter steel balls and a frequency of 20 Hz for a period of 4 min. The powder samples were packed into the appropriate sample holder to form a sintered disc.

Transmission Electron Microscopy was carried out with a High Resolution TEM Jeol Jem-2011 electron microscope (Jeol Ltd., Tokyo, Japan), with a resolution of 0.18 nm at 200 kV. The TEM samples, previously prepared by casting the nanocomposite into the specific mould described earlier, were cut with a Leica EM UC7 ultramicrotome (Leica Microsystems GmbH, Wetzlar, Germany) using a diamond knife (Diatome, Diatome Diamond Knives, Hatfield, UK), to give a thickness of approximately 50 nm. The ultrathin sections were left floating on water and were picked up and mounted on a 400 mesh carbon coated grid.

### 2.6. Mechanical Property Measurements

The storage modulus (*G'*), loss modulus (*G*″) and the mechanical loss factor (tan δ* = G"*/*G'*) were determined as a function of temperature and frequency by dynamic mechanical analysis (DMA) using the Mettler-Toledo model DMA861^e^ (Mettler Toledo AG Analytical, Schwerzenbach, Switzerland) in shear mode. Samples in the form of thin discs, approximately 6 mm diameter and 2 mm thickness, were machined from the moulded cylinders. DMA measurements were made over a range of frequencies (0.1, 0.3, 1, 3, 10, 30, 100 and 300 Hz) and at 2 °C/min heating rate over the temperature range from 50 to 285 °C.

The impact tests were performed at room temperature using a Zwick Izod impact tester 5110 (Zwick Roell, Ulm, Germany) according to ASTM D4508-05 (2008) on rectangular specimens, machined to 25 mm × 12 mm × 2.5 mm from the bulk moulded samples. The impact tester had a hammer of energy 0.545 J, and a minimum of eight specimens were tested for each composition.

After impact testing, the fracture surfaces of the impact samples were observed using a scanning electron microscope (SEM, Jeol 5610, Jeol Ltd., Tokyo, Japan) with an accelerating voltage of 15 kV and a spot size of 20, using secondary electron imaging. Before making the SEM observations, the fracture surfaces were sputtered with a thin layer of gold, between 10 and 15 nm thick, using a Baltec DSC005 sputter coater (Leica Microsystems GmbH, Wetzlar, Germany).

## 3. Results and Discussion

### 3.1. Thermal Analysis by DSC

**Figure 2:**
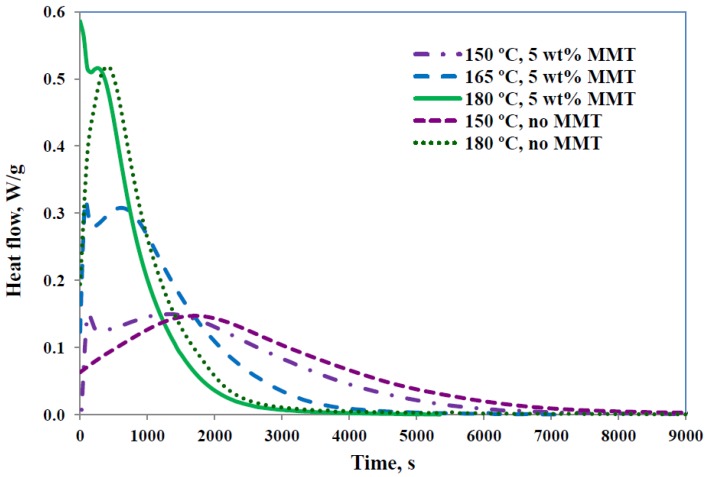
Isothermal cure of TGAP/MMT/DDS system: samples containing 5 wt% MMT at the temperatures of 150 °C (light purple, dash-dotted), 165 °C (blue, long dash), and 180 °C (light green, full); samples without MMT at the temperatures of 150 °C (dark purple, short dash) and 180 °C (dark green, dotted). Exothermic direction is upwards.

When prepared by the standard procedure described above, these nanocomposite systems using the tri-functional epoxy resin TGAP as the matrix material have previously been shown to be predisposed to exfoliate in view of the rapid intra-gallery reaction that takes place before the bulk cross-linking reaction [19,20,21]. This is illustrated in Figure 2 for TGAP/MMT/DDS samples with 5 wt% clay and cured isothermally at different temperatures, where the two overlapping reactions are clearly seen. The curing reactions for epoxy resins are complex, particularly in the presence of organically modified MMT, and have been discussed quite extensively elsewhere [17,22,25]. The several possible reactions include the reaction of the epoxide groups first with the primary amine and then with the secondary amine, and etherification catalysed by the tertiary amine and/or by the onium ion of the organically modified MMT. In the isothermal cure of the TGAP/DDS system without any clay, a single bell-shaped peak is found [26,27], shown in Figure 2 for the isothermal cure temperatures of 150 °C and 180 °C, which can be modelled as an autocatalytic reaction [20]. The rapid initial reaction that occurs in the same system with clay, TGAP/MMT/DDS, can therefore be attributed to the presence of the clay.

In order to maximise the degree of exfoliation of the clay layers, it is of interest that as much as possible of the first reaction should occur before the extra-gallery cross-linking reaction creates a rigid matrix around the clay particles. By deconvoluting these cure curves into two separate peaks, it was possible to determine the heat of reaction associated with each, and it was found that the magnitude of the first peak increased as the isothermal cure temperature increased, as also it did as the clay content was increased [20], this latter observation again supporting the hypothesis that the first peak is associated with an intra-gallery reaction. It would be anticipated, therefore, that the degree of exfoliation obtained in these nanocomposites would increase with the isothermal cure temperature, and this is indeed observed, as will be shown further below.

**Figure 3:**
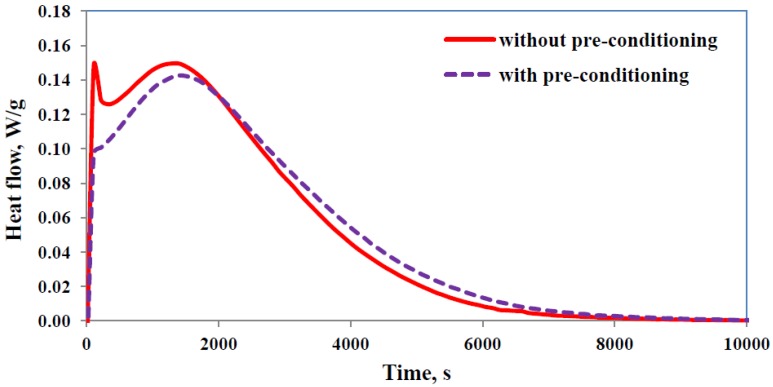
Comparison of isothermal cure curves at 150 °C for TGAP/MMT/DDS nanocomposites containing 5 wt% clay, with (purple curve, dashed line) and without (red curve, full line) pre-conditioning. Exothermic direction is upwards.

The intra-gallery reaction can also be promoted by the procedure of pre-conditioning, the second preparation procedure described above, which allows the homopolymerisation of the epoxy resin to take place within the clay galleries, catalysed by the onium ion of the organically modified clay. It is clear that a homopolymerisation reaction takes place during pre-conditioning, not only because the epoxy equivalent (understood as epoxy equivalent weight) and the glass transition temperature of the epoxy/clay mixture both increase, but also because the rapid first peak that was seen in the isothermal cure of TGAP/MMT/DDS nanocomposites did not appear if the resin-clay mixture had been pre-conditioned before the addition of the curing agent [19]. This is illustrated in Figure 3 for a sample that has been pre-conditioned for 109 days at room temperature before cure, compared with the cure of a fresh resin-clay mixture under the same conditions, where it can be seen that the initial peak has disappeared for the pre-conditioned sample. Although this procedure is slightly impractical, in that it requires storage of the resin-clay mixtures for relatively long times before the addition of the curing agent, it would be anticipated that pre-conditioning would result in an improved degree of exfoliation in the cured nanocomposite when compared with nanocomposites prepared by the standard procedure. This is because the intra-gallery reaction can proceed unhindered by any cross-linking reaction of the surrounding matrix. As will be shown below, the degree of exfoliation obtained by pre-conditioning the resin-clay mixture is indeed better than that for nanocomposites prepared by the standard procedure.

It might be argued that storage of the reactive resin, TGAP, for long periods of time, particularly at elevated temperatures, would lead to changes in the reaction kinetics even without any clay in the system. As a control experiment, the TGAP was stored for 14 days at 80 °C, and the glass transition temperature of the resin alone was determined before (−39.4 °C) and after storage (−37.7 °C). This small increase of 1.7 °C in *T*_g_ reflects a loss of epoxy groups, equivalent to a small increase in the epoxy equivalent [19], which would result in a small change in the reaction kinetics when cured with DDS. However, these changes are insignificant when compared with the changes that occur during pre-conditioning in the presence of organically modified MMT: storage at 80 °C of the TGAP/MMT system with 5 wt% MMT for 15 days or of the system with 2 wt% MMT for 13 days leads to changes in *T*_g_ of 27.1 °C and 23.7 °C, respectively, much larger than the 1.7 °C change for the resin without clay. Indeed, after storage for 2 years at room temperature the *T*_g_ of the TGAP increases to only −37.0 °C. It can be concluded that the pre-conditioning effects observed here are associated almost entirely with the presence of the clay.

**Figure 4:**
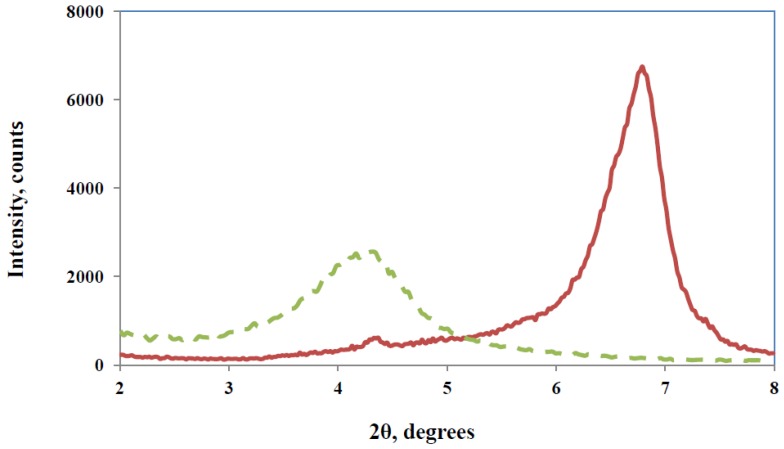
SAXS scattering diagrams for the organically modified MMT clay (green dashed line) and for the MMT intercalated with BF_3_·MEA (red solid line).

As an alternative to the somewhat impractical pre-conditioning procedure, we proposed earlier that the intra-gallery reaction could be promoted instead by incorporating an initiator of cationic homopolymerisation within the clay galleries before mixing the resin with the clay and adding the curing agent [21]. The preparation procedure described further above did indeed incorporate the initiator, BF_3_·MEA, into the clay galleries, as was demonstrated by SAXS, resulting in fact in a decrease in the layer *d*-spacing, from 2.1 nm for the organically modified MMT to 1.3 nm, as shown in Figure 4. This can probably be attributed to the interchange of the monoethylamine of the BF_3_ complex with the octadecyl ammonium salt of the organically modified MMT, and is therefore indicative of the presence of BF_3_·MEA within the clay galleries. Despite this reduction in the *d*-spacing, when the TGAP epoxy was mixed with this “initiated” clay, the resin intercalated into the clay galleries and caused the *d*-spacing to increase to 4.4 nm, a larger separation than the *d*-spacing of 3.5 nm found for the TGAP intercalated directly into the organically modified MMT [21].

It might be argued that there remain some traces of the solvent in the sample after the procedure used for the incorporation of the BF3·MEA into the clay galleries, and that this might affect the reaction kinetics. As a check on this possibility, TGAP/MMT/DDS samples with 5 wt% MMT and without any BF_3_·MEA were prepared by the solvent method [22,25] and then cured isothermally at 165 °C for 2 h. It transpires that there is very little difference in the reaction kinetics compared with the standard preparation procedure: the first, very rapid, reaction which is associated with intra-gallery homopolymerisation has a peak heat flow after about 30 s for the solvent-prepared sample in comparison with about 20 s for the standard sample, while the main cross-linking peak remains essentially unchanged, and the total heat of reaction is reduced from 651 J/g for the standard sample to 624 J/g for the solvent-prepared sample. These small differences could be attributed to the better dispersion of the clay in the resin which occurs for the solvent preparation method [22,25], which allows easier access of the resin to penetrate the clay galleries, and hence promotes homopolymerisation, with a corresponding decrease in the total heat of reaction. Accordingly, it is concluded that the solvent is indeed eliminated after the preparation procedure involving acetone, and that any small effects observed can be attribute to enhanced intra-gallery homopolymerisation.

It should be pointed out that it is unlikely that all the BF_3_·MEA enters the clay galleries, and hence that some may remain outside the galleries and be able to catalyse an extra-gallery homopolymerisation reaction. This is more likely to occur to a greater extent the higher is the proportion of BF_3_·MEA used. The consequence of this would be that, particularly for the higher BF_3_·MEA content, the extra-gallery reaction would lead to a more rigid matrix material surrounding the clay tactoids, which would inhibit the exfoliation process. This is a possible explanation for why, despite the anticipated greater extent of intra-gallery homopolymerisation reaction for the higher BF_3_·MEA content, improved exfoliation was not always observed by TEM for the BF_3_·MEA content of 1% compared with 0.5% [21]. The effect of the BF_3_·MEA content is discussed later in respect of the impact tests.

In order to examine further the possible effect of BF_3_·MEA on the cure of these nanocomposite systems, the TGAP/DDS system without any clay was cured isothermally after introducing 1 wt% of BF_3_·MEA in order to catalyse a homopolymerisation reaction. At the isothermal cure temperature of 150 °C, for which the TGAP/DDS system has an exotherm with a peak heat flow of less than 0.2 W/g occurring after more than 1000 s (see Figure 2, for example), the TGAP/DDS system with BF_3_·MEA has a peak heat flow at least an order of magnitude larger, and occurring almost instantaneously. Similar observations were made for cure at the much lower isothermal cure temperature of 120 °C. It can be concluded that a significant presence of BF_3_·MEA outside the gallery regions would accelerate the extra-gallery reaction to such an extent that the exfoliation of the clay layers by the intra-gallery reaction would be severely inhibited. The fact that this does not happen suggests that there is, in fact, hardly any BF_3_·MEA outside the clay galleries, in other words that the procedure adopted for introducing the initiator into the clay galleries has been very effective.

**Figure 5:**
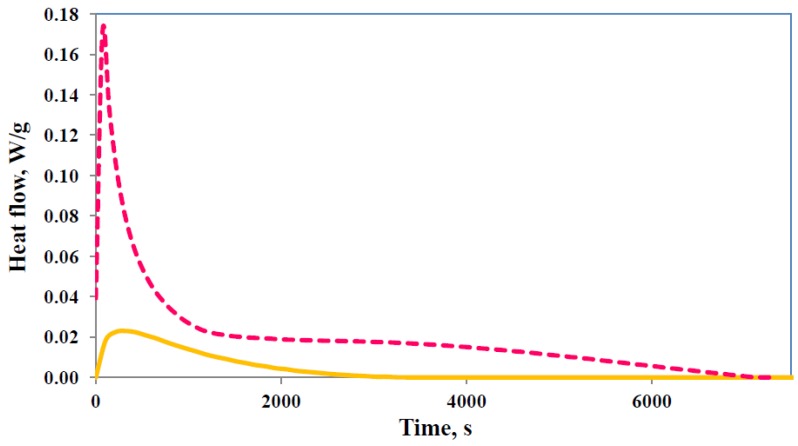
DSC scans for the isothermal cure of TGAP/DDS/MMT (5 wt%) with 1 wt% initiator (BF_3_·MEA) at different temperatures: 100 °C (orange, full line) and 120 °C (pink, dashed line). Exothermic direction is upwards.

The cure schedule for this system in which BF3·MEA is incorporated into the clay galleries was designed such that, in a first step at a relatively low temperature of 100 °C or 110 °C, only the cationically initiated homopolymerisation reaction between the clay layers would take place, the temperature being too low for any significant amount of cross-linking reaction to occur. In the second step, once the first reaction is complete, the system was isothermally cured at a higher temperature, for example 150 °C or 180 °C, and then finally post-cured at 210 °C. An illustration of the reaction taking place during the first cure step is shown in Figure 5. Here it can be seen that very little homopolymerisation occurs at 100 °C, whereas at 120 °C not only much more homopolymerisation but also some cross-linking occurs, seen as the broad shoulder at long times. Consequently, in order to maximise the amount of homopolymerisation that occurs, without any significant cross-linking reaction, the temperature for the first step in this procedure was usually selected as 110 °C, though some samples were prepared with 100 °C for this temperature (see Table 1).

### 3.2. Nanostructural Characterisation

**Figure 6:**
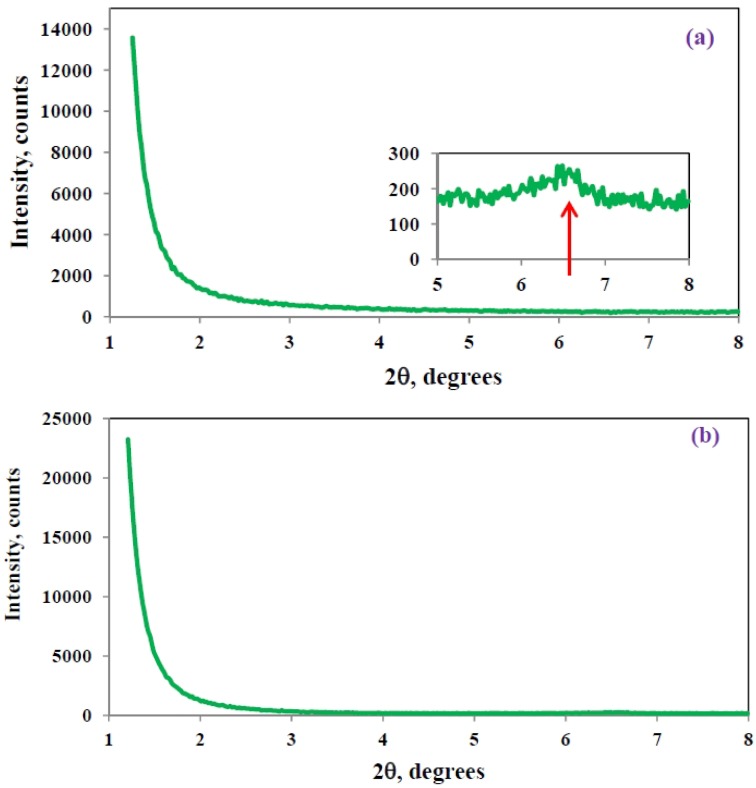
Typical SAXS diffractograms for nanocomposites: (**a**) for sample 5%, 180 °C; (**b**) for sample 2% + 0.5% BF_3_, 150 °C. The inset shows the small peak at 6.5 nm on an enlarged scale.

The SAXS diffractograms for all the samples prepared by the standard and pre-conditioning procedures showed no scattering peaks in the range of 2θ between 1.2° and 8°, indicating that there was no regular layer stacking with a *d*-spacing less than about 8 nm. An illustration is given in Figure 6a, for the particular case of a nanocomposite with 5 wt% MMT prepared by the standard procedure with an isothermal cure temperature of 180 °C, and a similarly featureless scattering pattern was found for the pre-conditioned samples. Consequently, nanocomposites prepared by these two procedures are clear candidates for presenting an exfoliated nanostructure, and should be studied by TEM, as will be shown below. For the samples prepared by the cationic initiation procedure, a very small scattering peak was often observed at 2θ close to 6.5°, corresponding to a *d*-spacing of approximately 1.3 nm. An example is given in Figure 6b for the particular case of a nanocomposite with 2 wt% MMT and 0.5 wt% BF_3_·MEA, cured isothermally first at 100 °C and then at 150 °C, where the small peak at 6.5° is shown on an enlarged scale in the inset. This *d*-spacing is the same as that for the MMT/BF_3_·MEA mixture before the intercalation of the epoxy resin into the clay galleries, and indicates that for a very small proportion of these “initiated” clay particles the epoxy resin does not intercalate into the clay galleries. This will be confirmed later by TEM.

**Figure 7:**
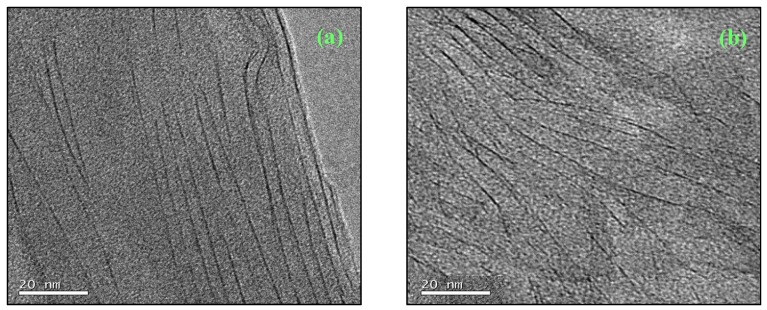
TEM micrographs for samples prepared by the standard procedure: (**a**) 5%, 150 °C and (**b**) 5%, 180 °C.

The TEM studies of the nanocomposites prepared by the different procedures revealed significant differences in the nanostructures, which can be correlated with the preparation procedures and with the thermal analysis of the cure process. As regards the standard preparation procedure, Figure 7 shows the nanostructure for two different isothermal cure temperatures, 150 °C and 180 °C, for samples with 5 wt% clay. It can be seen that both nanocomposites display a certain degree of exfoliation: even though some clay layers are still in register, the majority are separated by more than 8 nm, the maximum *d*-spacing detectable by SAXS, and are reasonably dispersed in the epoxy matrix. The separation and dispersion of the layers appears greater in the sample cured isothermally at the higher temperature, in agreement with the earlier observation from DSC that the higher isothermal cure temperature resulted in a greater contribution from the intra-gallery homopolymerisation reaction.

**Figure 8:**
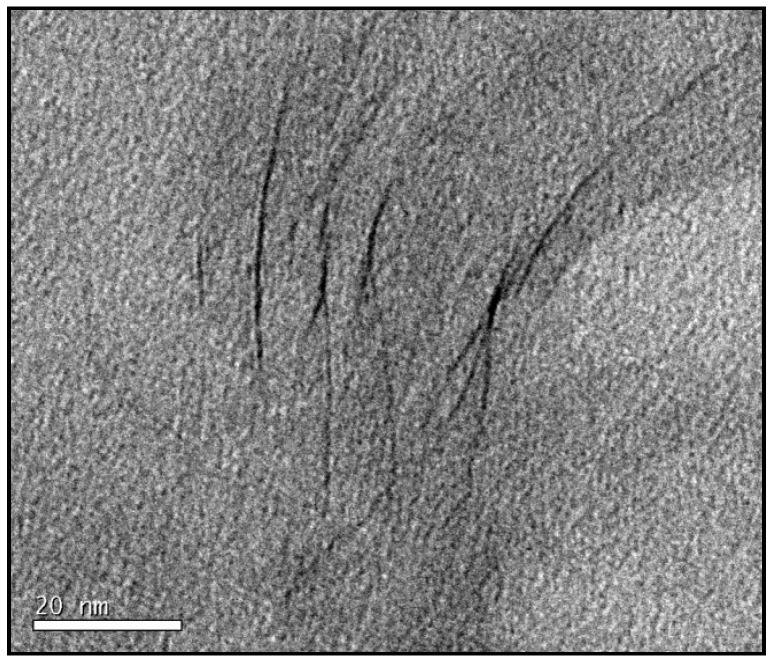
TEM micrograph for a sample with 2 wt% MMT, prepared by pre-conditioning at RT for 225 days.

For the pre-conditioning procedure, the TEM micrographs show, for example for a sample with 2 wt% clay pre-conditioned at RT for 225 days shown in Figure 8, that the degree of exfoliation was improved by pre-conditioning. Here it can be seen that there are very few clay layers visible, they are no longer in register, and that they are rather well separated and distributed throughout the matrix, indicating a significantly better degree of exfoliation than for samples prepared by the standard procedure, illustrated in Figure 7.

**Figure 9:**
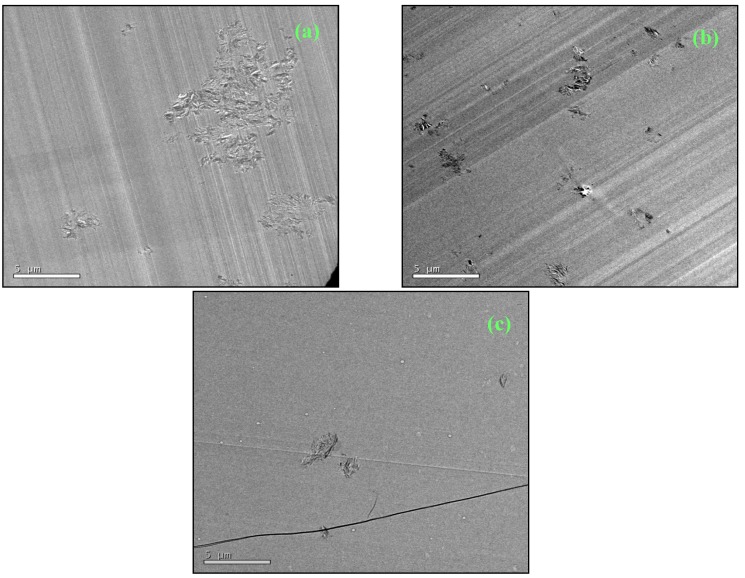
TEM micrographs at low magnification for samples with 5 wt% clay prepared by: (**a**) standard procedure, 180 °C; (**b**) pre-conditioning, 63 days at RT; and (**c**) cationic initiation with BF_3_·MEA, 5% + 0.5% BF_3_, 150 °C.

To illustrate the nanostructure of the samples prepared by the cationic initiation procedure, for which the extent of clay layer separation and the uniformity of their distribution is difficult to distinguish unequivocally from those for the pre-conditioned samples, we show first some TEM micrographs at lower magnification in order to indicate how the dispersion and size of the clay agglomerates is improved by this procedure, and how it compares also with the standard preparation procedure. Figure 9 shows the TEM micrographs at low magnification for samples with 5 wt% clay prepared by the three procedures: (a) standard; (b) pre-conditioning; and (c) cationic initiation. It can be seen that, for the standard procedure, some clay agglomerations remain after cure, the one shown here being of the order of 10 μm in size. For the pre-conditioned sample, Figure 9b, the agglomerations are much smaller, of the order of 2 μm in size, with several of these agglomerates dispersed throughout the matrix. In comparison, the cationic initiation procedure using BF_3_·MEA results in a significant reduction in the number of clay agglomerations, and generally that these agglomerates are also smaller; the largest agglomerate in Figure 9c is about 2 μm in size, and the few other agglomerates are much smaller.

**Figure 10:**
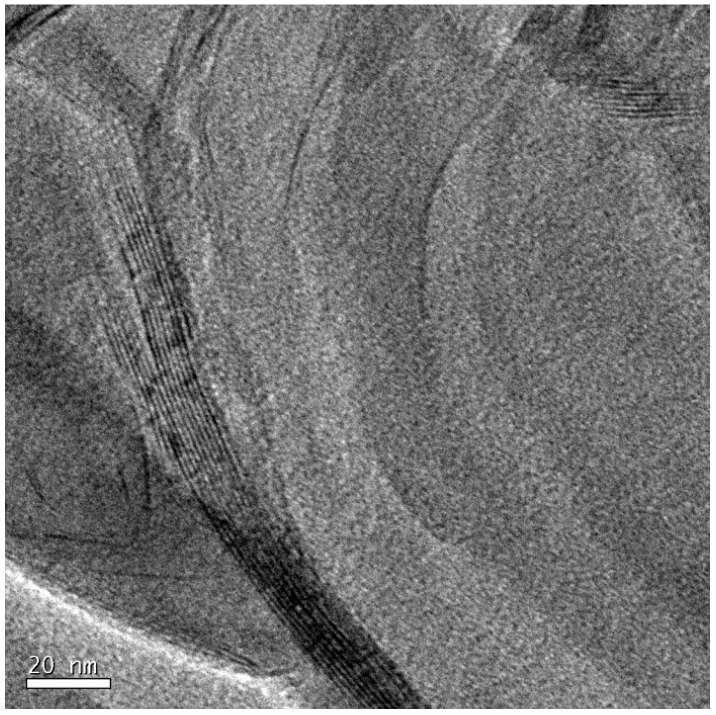
TEM micrograph at high magnification for sample with 5 wt% clay prepared by cationic initiation with 1% BF_3_·MEA and cured isothermally at 180 °C.

Although, as suggested above, the size and dispersion of the clay agglomerates is much improved by the BF_3_ preparation method, there still clearly remain some agglomerates, which in fact tend to be rather dense in comparison with the other preparation methods. This is illustrated by means of higher magnification TEM micrographs, such as that shown in Figure 10. Here, only a small part of the area towards the left of the micrograph contains stacked layers, with a high degree of exfoliation elsewhere. The layers which are stacked, however, have a *d*-spacing of only about 1.3 nm. These layers correspond to those discussed earlier, in the section on SAXS, where it was pointed out that for this preparation method the epoxy resin fails to intercalate into some of the clay tactoids in which the BF_3_·MEA has previously been intercalated, with the consequence that these layers remain with a separation of about 1.3 nm which they have before mixing this “cationically initiated” clay with the epoxy.

The identification and comparison of the nanostructures corresponding to the different preparation procedures by means of TEM, however, particularly at high magnification but also to a certain extent at low magnification, remains a somewhat subjective approach. This technique necessarily examines only a selected part of the overall sample, and even though the micrographs illustrated in Figure 7, Figure 8, Figure 9 and Figure 10 are considered to be representative and characteristic of the complete nanostructure, the observations made here should be supported by measurements of a property which depends on the global nanostructure. For these purposes, the dynamic mechanical properties and the impact resistance have both been determined and are discussed below.

### 3.3. Dynamic Mechanical Properties

**Figure 11:**
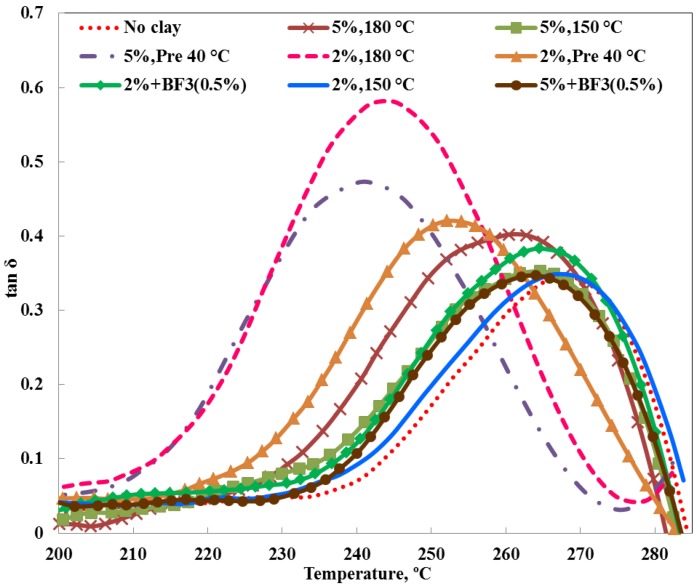
Tan δ as a function of temperature, at a frequency of 1 Hz, for different TGAP/clay nanocomposites, as indicated.

From the dynamic mechanical analysis (DMA) results, tan δ was determined for the nanocomposites prepared by the different procedures, and the dependence on temperature for a frequency of 1 Hz is shown in Figure 11. The maximum in tan δ, for example at a frequency of 1 Hz, is often taken as the dynamic glass transition temperature, *T*_g_, determined mechanically. More correctly, though, since this is frequency dependent, it is better to refer to this as the temperature of the α-relaxation, and so henceforth this temperature is denoted here as *T*_α_. Thus, it can be seen that *T*_α_ decreases for all the nanocomposites relative to the reference TGAP/DDS system without any clay. The decrease is relatively small, however, except for the two nanocomposites prepared by the pre-conditioning procedure and for sample 2%, 180 °C, this last appearing somewhat anomalous.

**Figure 12:**
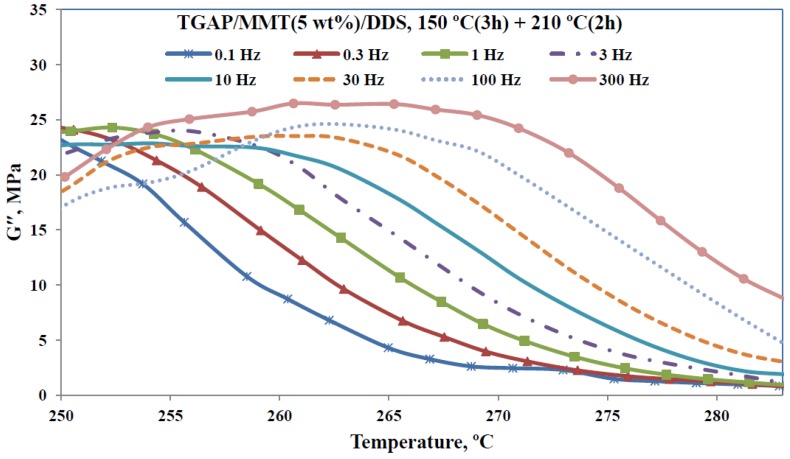
Loss modulus as a function of temperature, for the frequencies indicated in the insert, for the TGAP/MMT/DDS nanocomposite with 5 wt% clay content.

On the other hand, for filled systems such as the present PLS nanocomposites, the effect of the clay reinforcement will be more effective in the rubbery region than in the glassy region, and as a consequence there will be a corresponding effect on the location of the tan δ peak. It would therefore be interesting to examine also how the loss modulus depends on frequency and nanocomposite preparation method. Figure 12 shows the loss modulus curves for the nanocomposite sample TGAP/MMT(5)/DDS(52), for which the clay content is 5 wt%, the sample having been cured isothermally at 150 °C and then post-cured at 210 °C.

From the frequency dependence of the tan δ peaks in Figure 11 for each nanocomposite, over the full range of frequencies studied, the apparent activation energy for the α-relaxation can be determined. There is considerable variation with the different preparation procedures for the nanocomposites, but it does not appear to be systematic. In fact, using a uniform probability plot, the values of the activation energy are acceptably described by a uniform distribution. Based on the maximum likelihood estimation method, and a confidence limit of 95%, the interval of 800 ± 200 kJ/mol is obtained.

**Figure 13:**
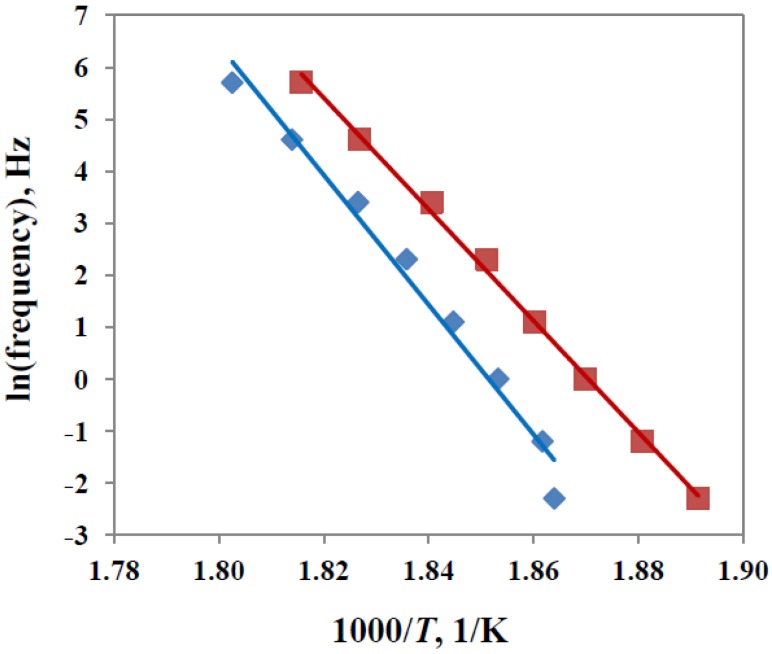
Plot of ln(frequency) *versus* inverse temperature for the frequency shift of the loss modulus curves, for two TGAP/MMT/DDS nanocomposite systems: red squares, 5 wt% clay content; blue rhombus, 2 wt% clay content.

Likewise, the activation energy can be determined from the loss modulus curves in Figure 12. For these curves, the peak was less well defined than was the high temperature flank of the relaxation, and hence the activation energy is determined from the temperature shift with frequency for this region. The results are plotted as ln(frequency) *versus* inverse temperature in Figure 13 for the sample for which the results are shown in Figure 12 as well as for another nanocomposite sample, TGAP/MMT(2)/DDS(52), for which the clay content is 2 wt%, this sample also having been cured isothermally at 150 °C and then post-cured at 210 °C. From the slope of the best-fit straight lines in Figure 13, the activation energies of 970 kJ/mol and 892 kJ/mol are obtained for the 2 wt% and 5 wt% samples, respectively. Both of these values fall within the 95% confidence interval for the value found from the tan δ peak, from which we conclude that, in this particular case, the activation energy is the same whether the determination of *T*_α_ is made from tan δ curves or from loss modulus data.

**Figure 14:**
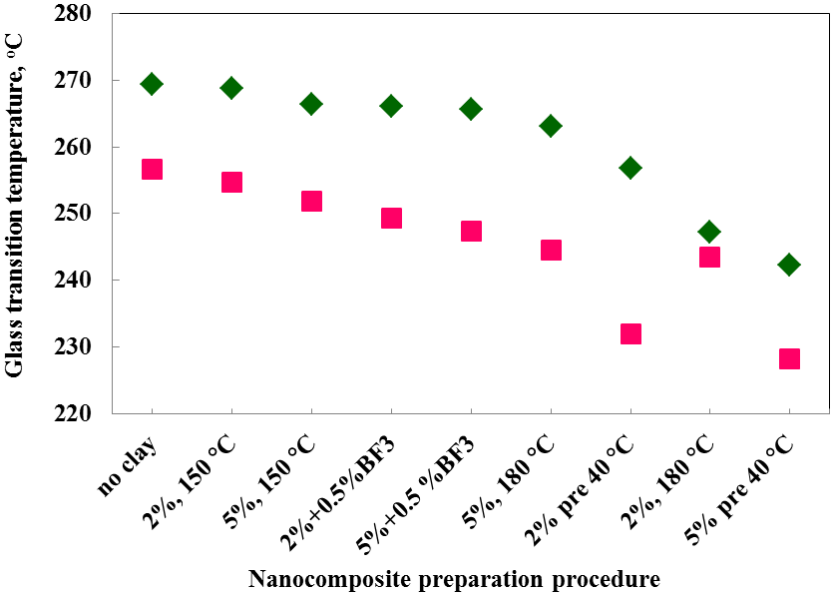
Variation of the dynamic glass transition temperature (or *T*_α_) with preparation procedure, obtained by two different techniques: DMA at 1 Hz (green rhombus) and TOPEM at 4 mHz (pink squares).

A certain decrease in *T*_α_ for the nanocomposites in comparison with the TGAP/DDS reference is not unexpected, as such a reduction on the addition of clay has often been observed before in other nanocomposite systems [7,17,28] for which the *T*_g_ was determined calorimetrically. In this respect, it is interesting to compare the values of *T*_α_ obtained by DMA with the dynamic *T*_g_ values obtained by a temperature modulated DSC technique, TOPEM, shown earlier to be convenient for the determination of *T*_g_ in these highly cross-linked systems [19,20,21,27]. The dynamic glass transition temperatures for the TOPEM measurements were taken from the step change in *c*_p0_, the quasi-static specific heat capacity, which can be associated with a frequency of about 4 mHz [29]. The comparison is shown in Figure 14.

It can be seen from Figure 14 that the DMA values for *T*_α_ are systematically greater than the TOPEM values, except for the result for sample 2%, 180 °C which again appears “anomalous”, as it did in Figure 11. The systematic difference between the mechanical and calorimetric α-relaxation temperatures is a consequence of the different frequencies at which *T*_α_ is determined. Over the whole range of nanocomposites in Figure 14, the average difference is about 16 °C, which corresponds to the same activation energy of 800 kJ/mol that was found earlier from the frequency dependence of the tan δ peaks. The slight decrease in *T*_α_ with the addition of clay is evident from this figure, as also is the more significant decrease for the preparation procedure involving pre-conditioning, which can be explained on the basis of the significant amounts of homopolymerisation which occur during pre-conditioning, resulting in a reduced cross-link density in the epoxy network.

Very little systematic correlation could be found between the storage modulus, evaluated in the glassy region at 50 °C, and the preparation procedure. The sample 2%, 180 °C prepared by the standard procedure again presents a somewhat anomalous behaviour, with a modulus more than 30% higher than the reference. In contrast, the nanocomposites prepared by the pre-conditioning procedure displayed lower values of storage modulus than the reference, particularly sample 2%, pre 40 °C, for which the storage modulus was reduced by almost 50%. This is presumably related to the greater extent of homopolymerisation in these nanocomposites, noted above also in respect of their *T*_α_ values, which results in a less cross-linked network structure. In the other nanocomposites, which all have the same modulus as the reference, within experimental error, it is likely that there is a competition between the effect of the clay, which is to increase the modulus, and the effect of homopolymerisation, occurring in all these systems to a greater or lesser extent, which is to reduce the modulus. As a consequence, no significant or systematic variations are observed.

### 3.4. Impact Tests

The dependence of many properties of epoxy/clay nanocomposites on the clay content has often been the subject of considerable discussion, and the fracture properties are no different in this respect. As a simple illustration, the following very different scenarios for the tensile strength of such nanocomposites relative to the unreinforced epoxy resin can be found in the literature: an increase for clay contents of 4 wt% [30] and 2.5 wt% [31]; essentially independent of clay content [32]; a slight decrease for clay contents up to 5 wt% [33] and 10 wt% [34]; a very small increase for a 2.5 wt% clay content, and thereafter a significant decrease [35]; a decrease up to a clay loading of 4 wt%, with a subsequent increase [36]; a decrease for a clay loading of 2 wt%, and then an increase up to a loading of 10 wt% [9]. It is evident that there are more factors than simply the clay content which influence the strength of these nanocomposites, and the same is true regarding their impact strength and toughness. For example, the topology of the epoxy matrix in respect of its ability to deform plastically, the interfacial strength between the clay nanoplatelets and the epoxy matrix, the extent of aggregation of the clay nanoparticles, as well as the degree to which the clay layers are exfoliated, are all factors which could play an important part to a greater or lesser extent. The following examples taken from the literature serve to illustrate the complexity of the situation.

Some authors believe that exfoliation is not the key to improved impact strength. For example, Fröhlich *et al*. [34] found—for anhydride cured nanocomposites based on DGEBA epoxy resin which were not exfoliated and had *d*-spacings of about 3 nm—that the toughness increased with clay content, though this increase was only modest, of the order of 60% in the value of *K*_Ic_ for clay contents up to 10 wt%. This increase in toughness for these intercalated nanocomposites, essentially equivalent to conventional microcomposites in view of their lack of exfoliation, was attributed to stress concentrations originated by the clay agglomerations resulting in shear yielding of the epoxy matrix. In earlier work, also on anhydride cured nanocomposites based on DGEBA epoxy resin, a similar conclusion had been reached [37]. Most of the nanocomposites in this work [37] had an intercalated nanostructure, but the few (hectorite-based) which were reportedly better exfoliated displayed a poorer toughness/stiffness balance; the TEM evidence for exfoliation in the hectorite-based system is, however, not convincing. The correlation between morphology and impact behaviour suggested that a “completely exfoliated” nanostructure predominantly influenced the stiffness, whereas the presence of intercalated clay agglomerations was the key to improved toughness, whereby nanovoids and shear yielding were promoted by the stress concentrating nanoparticles.

To a certain extent, this last interpretation is supported by the results of Miyagawa *et al.* [38] in a study of nanocomposites fabricated with biobased DGEBF epoxy resin and prepared by two methods, mechanical mixing and solvent preparation, in order to produce intercalated and exfoliated nanocomposites, respectively, each with 5 wt% clay. In agreement with the results of Zilg *et al.* [37], it was found that the modulus increased much more significantly in the exfoliated nanocomposites. On the other hand, it can also be seen that the impact strength was always higher for the exfoliated nanostructure, though the effect was only small. In later work, results were reported for amine cured DGEBA-based systems containing a reactive diluent and with varying clay contents [35]. The nanostructure consisted of both clay agglomerations and, reportedly, exfoliated clay nanoplatelets, giving what the authors refer to as an exfoliated/intercalated nanostructure. High magnification TEM shows, however, that, even outside the agglomerations, there are *d*-spacings as small as 4.32 nm, which is clearly not exfoliated. For these systems, the modulus increased significantly with increasing clay content while the Izod impact strength decreased markedly.

It is difficult to reconcile all the above results. Further support for the correlation of enhanced impact strength with increasing clay content in an intercalated nanostructure is afforded by the results of Ratna *et al.* [39]. Although this work is directed towards ternary epoxy/hyperbranched polymer/clay systems, the binary epoxy/clay systems are also studied, in which the DGEBA epoxy is cured with a diamine. No SAXS peaks are observed for the epoxy/clay nanocomposites with 5 wt% clay content, but TEM shows multiple layer stacking, with *d*-spacings between 9 and 10 nm. For these intercalated nanocomposites, the flexural modulus, flexural strength and the impact strength all increase on the addition of 5 wt% clay, the last by approximately 50%. The authors interpret the SEM micrographs of the fracture surfaces as showing evidence of massive shear deformation, attributed to shear yielding as a consequence of stress concentrations produced by the nanoparticles. On the other hand, Becker *et al.* [7] report a study of nanocomposites based upon epoxy resins of various functionalities, including DGEBA, in which the TEM images clearly show a large amount of clay layer stacking for all the systems investigated, implying essentially intercalated nanostructures, and yet for which there is no clear correlation between fracture toughness measured by *K*_Ic_ and clay content: for the tetra-functional resin there does appear to be a significant increase for both isothermal cure temperatures of 100 °C and 160 °C; for the tri-functional resin, TGAP, there is no significant effect, which is in direct contrast to what we will show below; and for the DGEBA resin there is a greater effect of the isothermal cure temperature than of the clay content, implying a contribution of the network structure in this case.

These results do not, however, provide any information about what might be the effect of an exfoliated nanostructure on the impact strength of these nanocomposites. Results for epoxy nanocomposites with, according to the authors, highly exfoliated clay were, however, presented by Wang *et al.* [33]. The comparison was made between nanocomposites prepared by conventional mixing and the slurry method, the latter giving a much better dispersion of the clay in the DGEBA epoxy: for the conventionally prepared nanocomposites, an intercalated nanostructure was observed in which there were clay aggregates with more than 50 ordered layers, whereas the slurry method produced supposedly “exfoliated” thin tactoids containing only a few clay layers, as evidenced by TEM micrographs in which the magnification (scale bar of 200 nm) is, unfortunately, too low to allow the identification of individual clay layers. Fracture toughness experiments show that, for the “exfoliated” nanocomposite, *K*_Ic_ increases slightly with clay content up to 5 wt%, with a marked peak at 2.5 wt% where the increase is almost a factor of 2, whereas there is very little change in *K*_Ic_ with clay content for the intercalated nanocomposite. The increase in toughness for the “exfoliated” nanocomposites was not accompanied by any shear yielding of the epoxy matrix; in contrast, the main toughening mechanisms were considered to be the formation of a large number of microcracks which are not coplanar and an increase in the fracture surface area due to crack deflection, both of which require a well dispersed and exfoliated nanostructure.

We present here our experimental results for the impact strength of the TGAP-based nanocomposites in the light of the above discussion. The different preparation procedures of these nanocomposites, presented schematically in Figure 1, have as their principal objective the achievement of an exfoliated nanostructure, and the results presented in the earlier sections of the present paper show the extent to which this has been achieved: the degree of exfoliation and the quality of the dispersion of the clay improve in the order standard procedure < preconditioning procedure < cationic initiation procedure. However, as was shown earlier, the different preparation procedures also result in differences in other aspects of the nanostructure and morphology, and in particular in the epoxy matrix network structure, as exemplified by the differences in the glass transition temperature. In the interpretation of the effect of preparation procedure on the impact strength, we attempt to take these aspects into consideration.

The values of the impact strength, together with the standard deviations, of the nanocomposites prepared by the different procedures are collected in Table 2. It can be seen that the impact strength of the nanocomposites is increased relative to the reference for all the preparation methods, except for two samples with 5 wt% clay content, namely 5%, 150 °C and 5%, 180 °C, which have approximately the same values as the reference. In fact, the impact strengths of all the nanocomposites with 5 wt% clay are less than those of the corresponding 2 wt% nanocomposite. This could be explained on the basis of the better dispersion of the clay agglomerates in the nanocomposites with the lower clay content, an effect observed previously for DGEBA-based nanocomposites in respect of other thermal properties [39,40]: the relatively large clay agglomerates in the 5 wt% nanocomposites would act as the sites of stress concentrations which would lead to premature failure in the epoxy matrix, which shows little sign of plastic deformation as will be seen shortly below. On the other hand, this runs contrary to the interpretations of some other studies, discussed above, where the stress concentrations resulting from the clay nanoparticles give rise to shear yielding of the matrix and improved toughness [34,37,39]. In view of this uncertainty regarding the effect of the clay content, we therefore concentrate first on the results for the 2 wt% nanocomposites in order to study the influence of the preparation procedure, and in particular the influence of the degree of exfoliation, on the impact strength.

For the nanocomposites prepared by the standard procedure, it can be seen that there is a slight increase in the impact strength as the isothermal cure temperature increases, though the change is not very significant in the light of the magnitude of the standard deviation. Nevertheless, this increase is consistent with the correlation of the impact strength with the degree of exfoliation: the earlier observations from both DSC and TEM showed, respectively, that for cure at 180 °C the contribution of the intra-gallery homopolymerisation reaction is greater than that for cure at 150 °C, and that the *d*-spacing is greater for isothermal cure at the higher temperature.

**Table 2:** Values of the impact strength for different TGAP/clay nanocomposites with 2 wt% and 5 wt% MMT. Reference: 1.40 ± 0.18 kJ/m^2^ (No clay).

Preparation procedure	Impact strength (kJ/m^2^)	
	2 wt% clay content	5 wt% clay content
150 °C	1.60 ± 0.20	1.34 ± 0.15
180 °C	1.70 ± 0.38	1.31 ± 0.10
pre 40 °C	2.10 ± 0.33	2.04 ± 0.32
+0.5% BF_3_, 125 °C	2.34 ± 0.24	1.54 ± 0.23
+0.5% BF_3_, 150 °C	2.50 ± 0.90	–
+0.5% BF_3_, 180 °C	2.17 ± 0.56	–
+1% BF_3_, 125 °C	2.91 ± 0.53	2.65 ± 0.22

A significantly greater increase in the impact energy, by 50% relative to the reference, is obtained for the preparation procedure involving pre-conditioning of the resin/clay mixture at 40 °C. This procedure ensures an extensive amount of intra-gallery homopolymerisation reaction, which leads to an improved degree of exfoliation as was shown in Figure 8, again in accordance with our interpretation of these impact results. In fact, almost the same value of impact energy is found for the 5 wt% nanocomposite also. This might be explained by the fact that pre-conditioning also improves the dispersion of the clay agglomerations, in particular reducing their size, which would imply that, after pre-conditioning, there would be less deterioration in the impact strength in the 5 wt% nanocomposites caused by the inferior clay agglomerate dispersion in comparison with the 2 wt% nanocomposites.

The greatest increase in impact energy for the 2 wt% nanocomposites, by over 100% relative to the reference for the best case, is obtained by the incorporation of BF_3_·MEA in a proportion of 1% between the clay galleries. This procedure promotes the intra-gallery reaction, which was shown earlier to lead to fewer and smaller aggregates in the cured nanocomposite (Figure 9). In fact, even the 5 wt% nanocomposite shows a significant increase in impact strength when prepared by the cationic initiation procedure, which demonstrates how effective this procedure is in improving the properties of these nanocomposites. These results are again consistent with a correlation of the impact strength with the degree of exfoliation of the clay.

We now consider briefly the possible effects on the impact strength of the changes in the epoxy matrix network topology as a consequence of the different preparation procedures. The results presented by Becker *et al.* [7] for nanocomposites based on DGEBA epoxy and cured initially, with diethyltoluenediamine, at two different isothermal temperatures, 100 °C and 160 °C, are interesting in this respect. In both cases, vitrification occurs during the first isothermal cure stage, and then a “fully cured” state is achieved after a post-cure schedule. These different cure schedules might be expected to give rise to different network topologies, but for the samples without any clay this is not reflected in any difference in the glass transition temperatures. On the other hand, for the nanocomposites with 7.5 wt% clay, curing initially at 100 °C resulted in a *T*_g_ significantly higher than that for the sample cured initially at 160 °C; this could be interpreted in terms of a greater degree of homopolymerisation, catalysed by the organically modified clay, occurring at the higher cure temperature, and hence a higher degree of exfoliation as this homopolymerisation will be concentrated within the clay galleries. This interpretation is consistent with the observed fracture toughness measurements, where *K*_Ic_ is consistently higher for the nanocomposites cured initially at the higher temperature, even though there is little effect of the magnitude of the clay content above 2.5 wt%. This interpretation leaves unresolved why *K*_Ic_ is also much higher for the resin without any clay cured at the higher temperature, but in respect to the effect of the clay it is consistent with our present arguments relating exfoliation and toughness.

**Figure 15:**
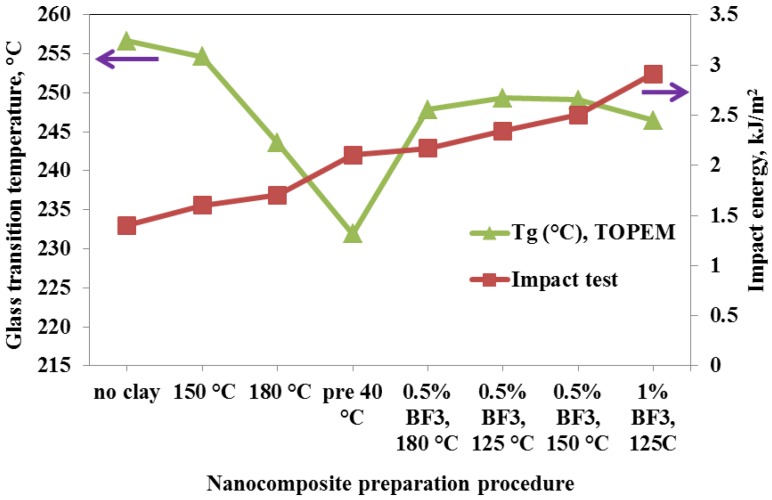
Variation of the dynamic glass transition temperature obtained by TOPEM (left hand axis) and impact energy (right hand axis) with preparation procedure for nanocomposites containing 2 wt% clay.

To examine more closely the relationship between the measured impact strength of the nanocomposites in the present work and their respective cross-linked network formations, both the dynamic glass transition temperature, determined calorimetrically by TOPEM, and the impact strength are plotted in Figure 15 as a function of the various preparation procedures. The preparation procedures are arranged in such a way that the impact strength increases from left to right, which can be interpreted in the following way. In order that the addition of clay to the epoxy resin will increase the impact strength, it is necessary for the clay to be exfoliated, and increasing degrees of exfoliation are observed (i) for increased isothermal curing temperatures, (ii) when the resin/clay mixture is pre-conditioned prior to curing; and (iii) when the BF_3_ MEA initiator is introduced into the clay galleries. At the same time, there is a trend for the dynamic glass transition temperature to decrease as the impact strength increases, even though there is clearly one result which does not fit this trend, namely that for the pre-conditioned sample. This trend could be understood in terms of an increasing amount of homopolymerisation taking place as the amount of intra-gallery reaction necessary for exfoliation increases, as the glass transition is lower for the homopolymerised network structure. An important corollary to be drawn from this is that the glass transition temperature should not be used as a measure of the quality of the nanostructure; in the present case, the increased degree of exfoliation for the samples with increased impact strength comes at the expense of a greater degree of homopolymerisation, which necessarily implies a reduction in the glass transition temperature.

**Figure 16:**
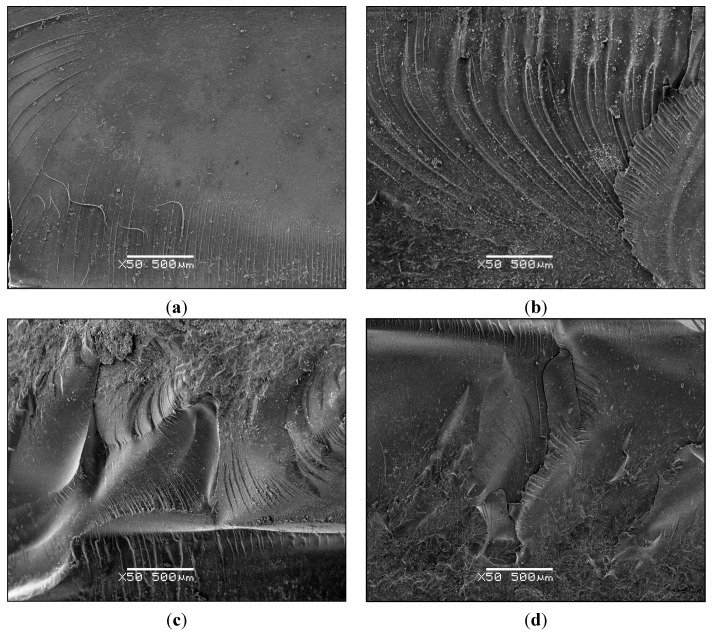
SEM micrographs of the fracture surfaces of 2 wt% MMT nanocomposite samples after impact testing: (**a**) reference, TGAP/DDS without any clay; (**b**) 2%, 150 °C; (**c**) 2%, pre 40 °C; (**d**) 2% + 1% BF_3_·MEA, 125 °C.

In the light of the above discussion we believe that the interpretation that the increased impact strength of these nanocomposites can be correlated with an improvement in the degree of exfoliation of the clay is consistent with the results presented here. This correlation leaves open, however, the question of the mechanism by which the impact strength is increased by improved exfoliation. As discussed above, the greater degree of exfoliation can be achieved by promoting epoxy homopolymerisation, which can simultaneously result in a reduced cross-link density of the epoxy, and hence a potentially more ductile matrix. Indeed, some matrix ductility was necessary to explain the improvements in toughness as a function of clay content reported by Mülhaupt and co-workers [34,37] and by Ratna *et al.* [39], as the energy absorbing mechanism was considered to be shear yielding initiated by stress concentrations resulting from the presence of nanoclay agglomerations. However, in the present work, examination of the fracture surfaces of the impact specimens by scanning electron microscopy (SEM) after the impact tests reveals very little evidence of plastic deformation. This is illustrated in Figure 16 for a selection of impact samples.

The reference sample TGAP/DDS without any clay (Figure 16a) shows the typical rather brittle fracture surface, with only a few striations where the crack velocity has increased. The amount of striations in Figure 16b, for the TGAP/MMT/DDS sample cured isothermally at 150 °C, is significantly greater, and the characteristic parabolic shape associated with the divergence of the crack from one plane to another can be seen. The overall appearance, though, is of an essentially planar fracture in both Figure 16a,b, and furthermore with little evidence of plastic deformation. On the other hand, the fracture surfaces seen in Figure 16c,d, for the pre-conditioned sample and the sample in which the BF_3_·MEA initiator was used, respectively, both show a generally much rougher surface. This is consistent with the significantly higher values of impact energy listed in Table 2 for these samples, and can be interpreted in terms of an increase in the fracture surface area as the mechanism responsible. In this respect, we adhere to the interpretation proposed by Wang *et al.* [33] for the toughening of “highly exfoliated” nanocomposites. These authors suggest that microcracks are formed in these nanocomposite systems in the regions close to the clay layers, and that these microcracks then propagate. So that they do not coalesce easily, they should not be coplanar, and so that many are initiated it is necessary for the clay layers to be separated, and not exist in large agglomerations. This scenario points to the need for an exfoliated nanostructure, and the increase in the impact energy comes from the increase in the fracture surface area due to a large number of microcracks as well as their deflection when the propagating crack meets a clay layer.

## 4. Conclusions

The cure kinetics, nanostructure and mechanical properties of tri-functional epoxy layered silicate nanocomposites, which are fabricated by three different preparation procedures, have been investigated. A clear relationship between features of the cure reaction, the nanostructure identified by SAXS and TEM, and the dynamic mechanical properties and the impact strength has been observed for each of the preparation methods, which permits the identification of the optimum procedure for achieving highly exfoliated nanocomposites. The crucial feature common to all the procedures is the separation of the intra-gallery homopolymerisation from the extra-gallery cross-linking reaction, and in particular the promotion of the intra-gallery reaction such that it occurs to a maximum extent and before the extra-gallery reaction. The intra-gallery reaction can be promoted by fabricating the nanocomposite by three distinct procedures: (a) by increasing the temperature of isothermal cure; (b) by pre-conditioning the resin/clay mixture before adding the curing agent; and (c) by incorporating an initiator of cationic homopolymerisation into the clay galleries. Each of these procedures improves the degree of exfoliation as identified by TEM, the extent of improvement being best for procedure (c) and least for procedure (a). These improvements in the nanostructure could be anticipated from the cure kinetics observed by DSC, and correlate with the improvements in the impact strength of the cured nanocomposites. It is concluded that the incorporation of an initiator of cationic homopolymerisation into the clay galleries in order to promote the intra-gallery homopolymerisation reaction is an effective procedure for achieving high degrees of exfoliation in the cured nanocomposite.
